# Coral Sr/Ca records provide realistic representation of eastern Indian Ocean cooling during extreme positive Indian Ocean Dipole events

**DOI:** 10.1038/s41598-022-14617-9

**Published:** 2022-06-23

**Authors:** Miriam Pfeiffer, Takaaki Konabe Watanabe, Hideko Takayanagi, Sri Yudawati Cahyarini, Dieter Garbe-Schönberg, Tsuyoshi Watanabe

**Affiliations:** 1 Institute of Geosciences, University of Kiel, 24118 Kiel, Germany; 2 KIKAI Institute for Coral Reef Sciences, Kikai Town, Kagoshima, 891-6151 Japan; 3 Institute of Geology and Paleontology, Graduate School of Science, Tohoku University, Sendai, 980-8578 Japan; 4 Department of Physics and Earth Sciences, Jacobs University Bremen, 28759 Bremen, Germany; 5 Department of Natural History Sciences, Faculty of Science, Hokkaido University, Sapporo, 060-0810 Japan; 6 Res. Group of Paleoclimate & Paleoenvironment, Res.Centre for Climate and Atmosphere, National Research and Innovation Agency Republic of Indonesia (BRIN), Bandung, Indonesia

**Keywords:** Climate sciences, Palaeoclimate

## Abstract

Extreme positive Indian Ocean Dipole (pIOD) events are amplified by non-linear ocean–atmosphere interactions and are characterized by pronounced cooling in the eastern equatorial Indian Ocean. These non-linear feedbacks are not adequately represented in historical products of sea surface temperatures that underestimate the magnitude of extreme pIOD events. Here, we present a sea surface temperature (SST) reconstruction based on monthly coral Sr/Ca ratios measured in two coral cores from Enggano Island (Indonesia), that lies in the eastern pole of the IOD. The coral SST reconstruction extends from 1930 to 2008 and captures the magnitude of cooling during extreme pIOD events as shown in recent satellite and reanalysis data of SST that include ocean dynamics. The corals indicate that the 1961 pIOD event was at least as severe as the 1997 event, while the 1963 pIOD was more comparable to the 2006 event. The magnitude 1967 pIOD is difficult to assess at present due to poor replication between coral cores, and may be comparable to either 1997 or 2006. Cooling during the 1972 pIOD was short-lived and followed by pronounced warming, as seen in the moderate pIOD event of 1982. A combination of coral SST reconstructions and an extension of new reanalysis products of SST to historical time scales could help to better assess the severity and impact of past pIOD events such as the ones seen in the 1960s.

## Introduction

**Figure 1:**
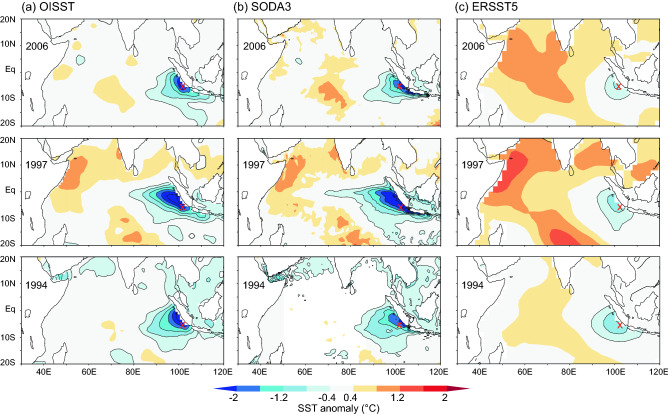
September–November mean SST anomalies during the extreme pIOD events of 2006, 1997 and 1994 as depicted in satellite SSTs (OISST), reanalysis SST (SODA3) and historical SST products (ERSST5). Charts created using the knmi climate explorer (https://climexp.knmi.nl/start.cgi; data accessed 11/03/2022). See text for discussion.

The Indian Ocean Dipole (IOD) describes an aperiodic oscillation of sea surface temperatures (SST) in the equatorial Indian Ocean^1,2^. A positive IOD (pIOD) causes upwelling and cooling in the eastern equatorial Indian Ocean, off the coast of Java and Sumatra (Fig. 1), and droughts in adjacent land areas of Indonesia and Australia^1–3^. The western and central Indian Ocean shows moderate warming, which nevertheless causes above-average precipitation and flooding in the central Indian Ocean and equatorial East Africa due to the warm mean SSTs in the region^2,4–6^ (Fig. 1). The negative phase of the IOD (nIOD) causes opposite conditions, with warmer water and greater than average precipitation in the eastern Indian Ocean, and cooler and drier conditions in the west^1,2^.

A well-known feature of the IOD index is its skewness, as pIOD events may grow much larger than negative IOD events (nIOD), so that the IOD is positively skewed^7,8^. The positive skewness of the IOD index reflects the negative skewness of SST in the eastern pole of the IOD (IODE; 90°E–110°E, 10°S-Eq.), as western Indian Ocean SSTs show only a weak positive skewness (Fig. S1)^9,10^. The negative skewness of IODE SSTs is caused by a positive Bjerkness feedback involving the SST response to the depth of the thermocline in the eastern Indian Ocean: cold IODE SST anomalies lead to a zonal SST gradient that drives an easterly wind anomaly in the equatorial Indian Ocean, which further shoals the thermocline and reinforces the cold SST anomalies in the IODE region^7,8,11^. Due to this asymmetry, pIOD events tend to have stronger cold sea surface temperature anomalies over the eastern pole of the IOD than warm SST anomalies during nIOD events^8^. In addition, pIOD events display strong inter‐event differences, with extreme events dominated by westward‐extended strong cold anomalies along the equator (Fig. 1), and moderate events with weakened cooling confined to the region off Sumatra‐Java^11,12^. This is due to non-linear zonal and vertical advection of cold water during extreme pIOD events in the IODE region, which occurs in addition to the Bjerkness feedback^11^. Climate models suggest that extreme pIOD events may increase in frequency under greenhouse warming, due to the faster warming of the western Indian Ocean that favors nonlinear advection in the eastern equatorial Indian Ocean^12,13^.

Extreme pIOD events have particularly devastating impacts in the countries surrounding the Indian Ocean. For example, the extreme pIOD event of 2019 caused extreme droughts and bushfires over Indonesia and Australia, as well as severe flooding in equatorial East Africa followed by plagues of locusts^14^. The extreme pIOD event of 1997 led to large-scale warming in the western Indian Ocean (Fig. 1), which caused extensive coral bleaching and mass mortality^15^. Given the severe socio-economic impacts of extreme pIOD events, their adequate representation in instrumental SST products is of primary importance. However, the magnitude of cooling indicated in the IODE region during extreme pIOD events varies between temperature products (Fig. 1). In particular, historical SST products based on interpolation from sparse historical observations underestimate the magnitude of the cooling, while reanalysis products that include subsurface oceanographic processes and their non-linear dynamics capture the magnitude of extreme pIODs more realistically^11^. However, beyond the start of the satellite era in 1982, the IOD index is based on historical SST products^16^ that do not adequately capture the cooling in the IODE region^11^. Therefore, the magnitude of extreme pIOD events prior 1982 is difficult to assess.

Tropical corals can be used to reconstruct past changes in SST at monthly resolution by measuring the Sr/Ca ratios in skeletal aragonite, which have been shown to be a very reliable paleothermometer and provide independent constraints on historical SST observations^5,17,18^. The high temporal resolution of coral proxy data allows the reconstruction of seasonal climatic phenomena such as the IOD^19–22^, and as coral Sr/Ca ratios are inversely correlated to ambient water temperatures during the corals’ growth, they should mirror SST variability in the IODE region, including its non-linearity. To date, however, most coral reconstructions from the IODE region focused on coral δ^18^O^21^, which reflects a combination of SST and δ^18^O seawater and does not allow quantitative estimates of SST anomalies. In a recent study, 40-year coral Sr/Ca record from Enggano island, located off the coast of Sumatra (Indonesia) has been shown to track IODE SST variability^22^. Here, we extend this record back until 1930, present a replication core and reconstruct SST variability in the IODE region from Enggano coral Sr/Ca ratios. We use a Monte Carlo simulation to estimate the uncertainties of the multi-colonial reconstruction^23,24^. The coral SST reconstruction is compared with various SST products (satellite observations, reanalysis products that include ocean dynamics and historical products based on statistical interpolation from sparse data), with a particular focus on extreme pIOD events. We will show that despite their uncertainties, the corals provide a better indicator of IOD-induced cooling in the eastern equatorial Indian Ocean than historical SST products interpolated from sparse data.

## Results and discussion

### Coral Sr/Ca data, SST conversion and uncertainties

**Figure 2:**
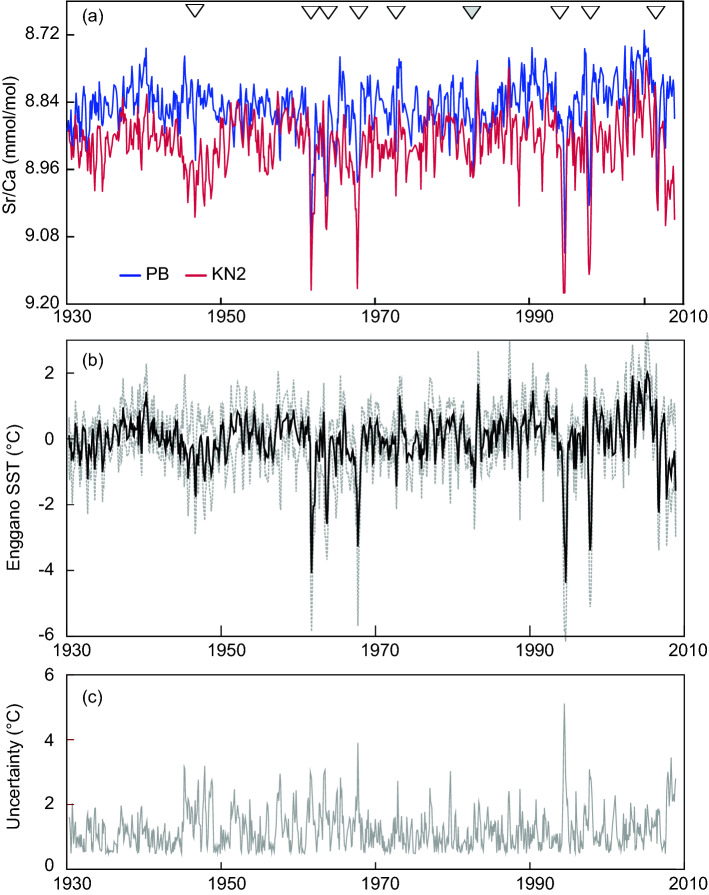
(**a**) Monthly coral Sr/Ca records of KN2 and PB. (**b**) Enggano coral SST record estimated from KN2 and PB (black solid line). Uncertainty envelopes (95% confidence levels, gray dashed line) are estimated via a Monte Carlo approach^23,24^. (**c**) Uncertainty of the Enggano SST record in °C. Arrows in (**a**) mark extreme pIOD events of 2006, 1997 and 1994 discussed in^11^, the El Niño year of 1982 (grey arrow), and the true pIOD events of 1972, 1967, 1963, 1961 and 1946 listed in^28^ (from right to left).

The two modern coral Sr/Ca records (KN2 and PB) extend from 1930 to 2008 and derive from different sites at Enggano Island (Fig. S3; Fig. 2). The fidelity of KN2, PB and a composite record (the arithmetic means of KN2 and PB, hereafter referred to as ‘Enggano’) was evaluated by an Ordinary Least Squares (OLS) regression with OISST, which is based on satellite data and the best available instrumental SST product to characterize IODE SST variability^11^. The calibration period is limited by the availability of satellite data and extends from 1982 to 2008. Table 1 provides a summary of the OLS equations obtained for monthly and annual mean data. The KN2 and PB coral Sr/Ca records are each highly correlated with OISST and the slopes of the Sr/Ca-SST regressions fall in the range of previously published estimates^17,23^. As expected, the Enggano Sr/Ca record has the highest correlation with OISST (r = 0.81, p ≪ 0.01, n = 26), so we will mainly focus on this record for further analysis.

**Table 1:** Linear regression equation and correlation coefficient between annual mean and monthly coral Sr/Ca and NOAA OISSTv2 centered at Enggano Island (5°S, 102°E) for the time period 1982–2008 and the mean equation from^17^ for comparison.

Coral core	Regression equation	r (r^2^)	p	σ	n
	[Sr/Ca = slope(± standard error) x SST + intercept(± standard error)]				
**Annua**l					
PB	Sr/Ca = − 0.061(± 0.01) × SST + 10.607(± 0.30)	0.76 (0.58)	≪ 0.01	0.014	26
KN2	Sr/Ca = − 0.075(± 0.01) × SST + 11.077(± 0.37)	0.76(0.58)	≪ 0.01	0.024	26
Enggano	Sr/Ca = − 0.068(± 0.01) × SST + 10.821(± 0.28)	0.81(0.66)	≪ 0.01	0.015	26
**Monthly**					
PB	Sr/Ca = − 0.045(± 0.002) × SST + 10.119(± 0.07)	− 0.71(0.50)	≪ 0.01	0.038	322
KN2	Sr/Ca = − 0.054(± 0.003) x SST + 10.257(± 0.09)	− 0.71(0.50)	≪ 0.01	0.046	322
Enggano	Sr/Ca = − 0.047(± 0.003) × SST + 10.208(± 0.08)	− 0.67(0.45)	≪ 0.01	0.035	322
Mean Equation (Corrège, 2006)	Sr/Ca = − 0.0607 × SST + 10.553				

r(r^2^) is the correlation coefficient, p is the p-value and σ is the standard deviation of the regression.

The Enggano Sr/Ca record was centered to its mean and converted to SST units using the average Sr/Ca SST relationship of − 0.06 mmol/mol per 1 °C^17,23^. Its uncertainties were calculated in °C following^23,24^ and include the analytical error of monthly coral Sr/Ca determinations, the slope uncertainties of the Sr/Ca paleothermometer^23^, and time-varying differences between the monthly Sr/Ca records of KN2 and PB^24^ (see “Methods” for further details) (Fig. 2). Note that the uncertainties of the Enggano coral SST record may exceed 2 °C (95% Confidence Intervals) during extreme pIOD events (the calibration slope uncertainty is a factor in the error term, so that larger deviations from the mean have larger uncertainties, see^23^) and in intervals where the KN2 and PB Sr/Ca records show large deviations, i.e. after 2006 and between 1945 and 1951 (Fig. 2). It is unclear what the causes are for the latter. Potential candidates include local climatic factors and so-called vital effects that may affect the incorporation of Sr in coral skeletal aragonite, or a combination of both.

### Cooling during extreme pIOD events in coral and instrumental data: satellite era

In the satellite era starting in 1982, four extreme pIOD events occurred: 1994, 1997, 2006 and 2019 (the latter is not covered by the coral Sr/Ca records as the corals were drilled in 2008). Figure 1 compares the spatial pattern of SST anomalies in the tropical Indian Ocean during the extreme pIOD events of 1994, 1997 and 2006, as represented in satellite SST (OISST), reanalysis SST including ocean dynamics (SODA3) and historical products based on spatial interpolation (ERSST5). While OISST and SODA3 indicate cooling > − 2 °C off Sumatra and a westward extension of cold anomalies beyond 90°E, historical products such as ERSST5 show much weaker cooling (< − 1.5 °C) limited to the region east of 90°E. This results in a lower standard deviation and lower skewness of IODE SSTs in historical SST products compared to satellite and reanalysis SSTs (Figs. S1, S2). Products such as ERSST5 and HadISST1 that are created using spatial interpolation techniques to fill in gaps between sporadic observations blur SST anomalies and under-represent oceanic processes that are critical for the development of extreme pIOD events, and this can be seen even in the most recent periods covered by satellite observations [see^11^ for an extensive analysis and discussion].

**Figure 3:**
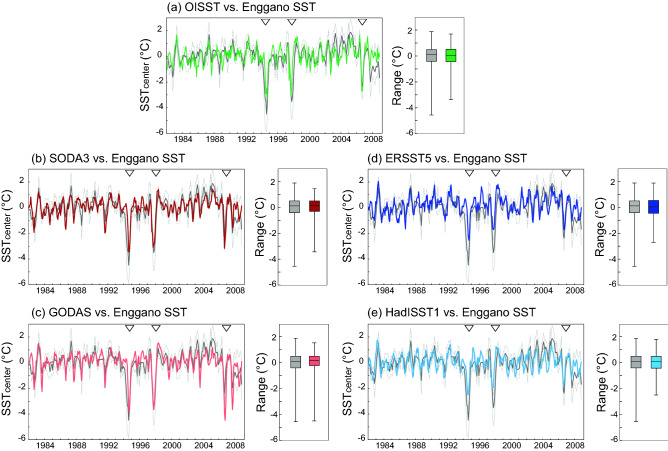
Monthly instrumental SSTs from various products and their SST range (boxplots showing median, interquartile range and maxima/minima as whiskers) compared with the monthly Enggano SST record (in grey) for the time period of 1982–2008. (**a**) OISST (green), (**b**) SODA3 SST (dark red), (**c**) GODAS SST (light red), (**d**) ERSST5 (dark blue), (**e**) HadISST1 (light blue). Arrows mark extreme pIOD events of 2006, 1997 and 1994 discussed in^11^. All time series have been centred to their mean.

**Figure 4:**
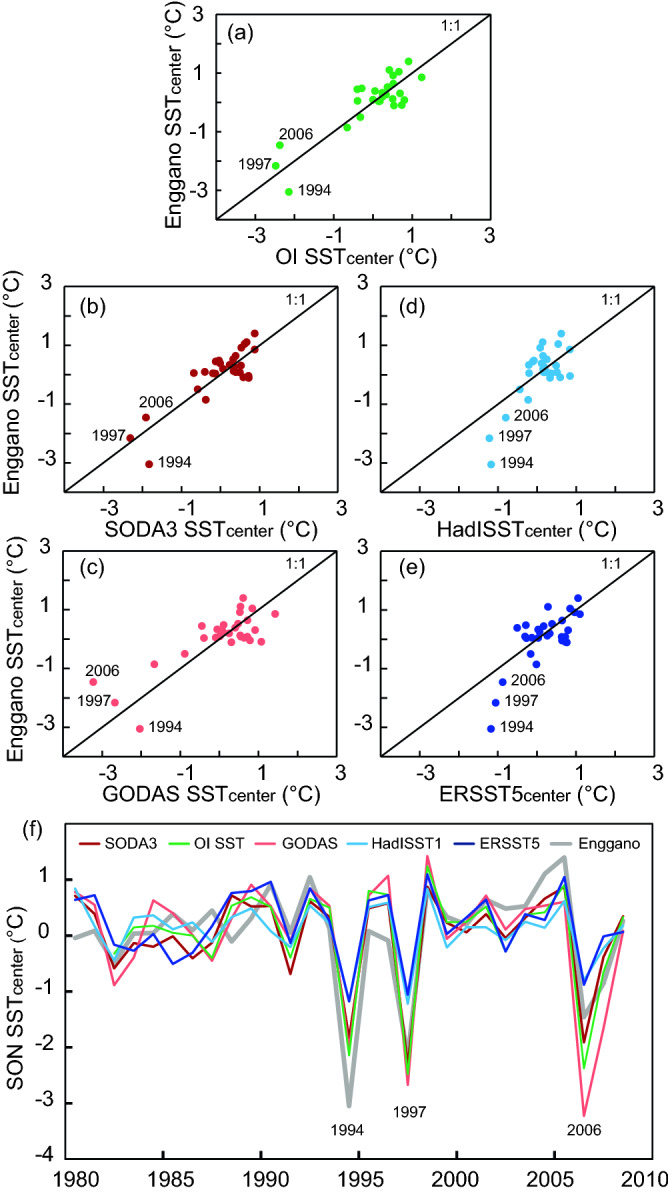
Scatter plots (**a**–**e**) and time series (**f**) of September–November SSTs from various SST products compared with Enggano coral SSTs for the time period of 1982–2008. (**a**) OISST vs. Enggano SST (green), (**b**) SODA3 vs. Enggano SST (dark red), (**c**) GODAS vs. Enggano SST (light red), (**d**) ERSST5 vs. Enggano SST (dark blue), (**e**) HadISST1 vs. Enggano SST (light blue). Note the cool anomalies during the extreme pIOD events in 2006, 1997 and 1994, which are underrepresented in HadISST1 and ERSST5. All time series have been centred to their mean.

Enggano Island is located in a region where (a) cold anomalies during extreme pIOD events exceed − 2 °C, and (b) discrepancies between satellite, reanalysis and interpolation-based products of SST are large (Fig. 1, Figs. S1, S2, S3). Figure 3 compares monthly time series of the Enggano SST record with satellite SST (OISST), reanalysis products of SST that incorporate ocean dynamics (SODA3 and GODAS), and historical products of SST that use statistical interpolation methods (ERSST5, HadISST1) to produce spatially complete fields of SST. The coral SST reconstruction, OI SST, SODA3 and GODAS show pronounced cooling during the extreme pIOD events of 2006, 1997 and 1994. While in these years cooling is also seen in ERSST5 and HadISST1, its magnitude is systematically underestimated (as shown by^11^). Importantly, the Enggano coral SST record is consistent both with satellite SST and with reanalysis SSTs that include ocean dynamics, whose non-linearity causes the pronounced cooling during extreme pIOD events^11,12^. This is also seen in time series of September–November average SSTs, i.e. the season when pIOD cooling peaks in the eastern equatorial Indian Ocean (Fig. 4). In scatter plots that compare Enggano coral SSTs with satellite and reanalysis SSTs, all data pairs are distributed along a line with an intercept of 0 and a slope of 1. This includes the extreme pIOD events of 2006, 1997 and 1994. In contrast, extreme pIOD events plot below this line in scatter plots comparing coral SST with ERSST5 and HadISST1, as these products underestimate the cooling. This is seen despite moderate to high linear correlations between Enggano Sr/Ca and these SST products (Tables S1, S2).

### Cooling during extreme pIOD events in coral and instrumental data: historical period

**Figure 5:**
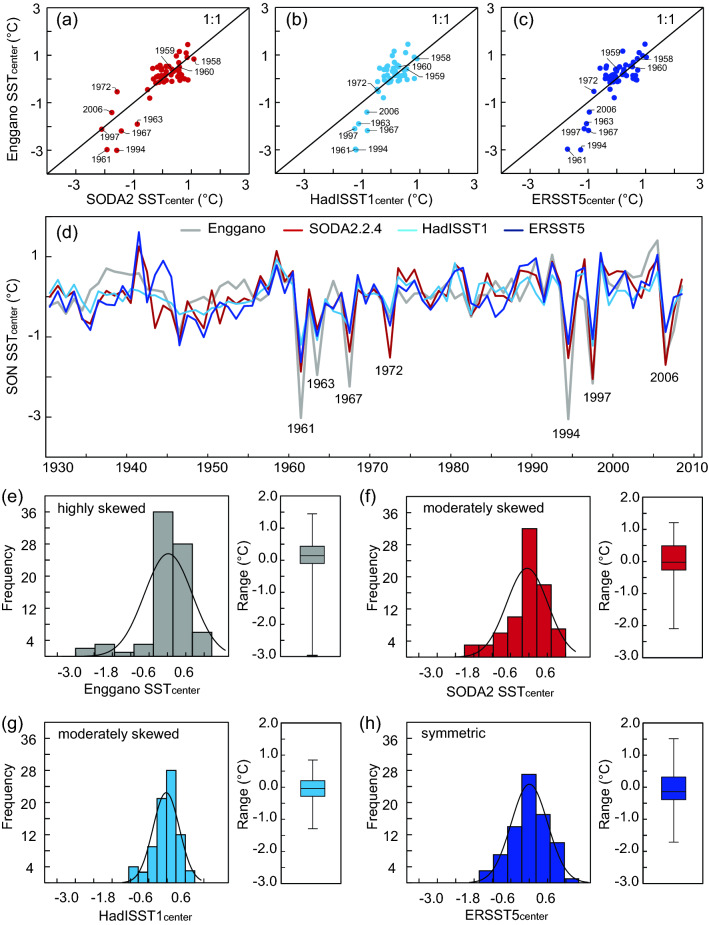
Scatter plots (**a**–**c**), time series (**d**), histograms and boxplots (**e**–**h**) of September–November SSTs from various SST products compared with Enggano coral SSTs for the time period of 1958–2008 (**a**–**c**) and 1930–2008 (**d**–**h**). Note the extreme cooling during the pIOD events of 2006, 1997, 1994, 1967, 1963 and 1961, which is underrepresented in HadISST1 and ERSST5 (**b**–**d**) and the highly skewed distribution of the Enggano SSTs compared to the moderately skewed (SODA2, HadISST1) and symmetric (ERSST5) distribution of historical products (**e**–**h**). Boxplots show median, interquartile range and maxima/minima (whiskers) of the datasets. All time series have been centred to their mean.

Beyond 1980, the only reanalysis product of SST available is SODA2.2.4, so we include it in our comparison although it has been superseded by SODA3. Figure 5 compares September–November mean Enggano SSTs with SODA2.2.4, ERSST5 and HadISST1 for the time period of 1930–2008. In this period, true pIOD events occurred in 1961, 1963, 1967, 1972, 1994, 1997 and 2006^11,26^. The SODA2.2.4 SSTs show a larger variability and a better match with Enggano coral SSTs than ERSST5 and HadISST1. In Scatter plots, true pIOD events plot close to a line with an intercept of 0 and a slope of 1, although the match is not quite as good as in the satellite era (note that the atmospheric reanalysis used in the creation of SODA 2.2.4 includes monthly SSTs from the HadISST1 data set^25^ that tends to underestimate pIOD variability). The largest mismatch between the coral SSTs and SODA2.2.4 is seen in 1972, where SODA shows cooling exceeding the extreme pIOD event of 1994, while the coral SSTs indicate weaker cooling in line with ERSST5 and HadISST1. In fact, the Enggano coral record indicates a short-lived cooling episode in the boreal fall season of 1972 followed by pronounced warming (Fig. 2), in response to the strong El Niño event that occurred in that year, which led to basin-scale Indian Ocean warming and above average precipitation in boreal winter and spring^5,22,26^. According to the Enggano SST record, the 1972 pIOD event would therefore classify as a moderate pIOD event.

In the ERSST5 and HadISST1 products, all true pIOD events (1961, 1963, 1967, 1994, 1997 and 2006) except 1972 again plot below a line with an intercept of 0 and a slope of 1, suggesting that these products do not adequately capture the non-linearity of these events. Interestingly, this does not apply to the extreme nIOD events that occurred in 1958, 1959 and 1960^27,28^, underlining that while non-linear feedbacks amplify extreme pIOD events, nIOD events are not amplified, and potentially dampened by ocean–atmosphere interactions^11,12^. September–November Enggano SSTs are highly skewed towards negative values, followed by SODA2.2.4 and HadISST1 SSTs that are moderately skewed, while ERSST5 SSTs are close to symmetric (Fig. 5, Table 2). As Enggano SSTs agree better with satellite data than ERSST5 and HadISST1, we believe that it provides a more realistic estimate of September–November SST variability in the IODE region beyond 1982.

**Table 2:** Basic statistics of September–November mean coral SSTs and various historical SST products centered at Enggano Island (5°S, 102°E) for the time period 1930–2008.

	Enggano (95% CI)	SODA2.2.4 (95% CI)	HadISST1 (95% CI)	ERSST5 (95% CI)
Min	− 3	− 2.11	− 1.25	− 1.71
Max	1.45	1.2	0.9	1.54
St. Dev	0.79 (0.45/0.98)	0.68 (0.53/0.79)	0.43 (0.34/0.51)	0.6 (0.49/0.69)
Skewness	− 2.07 (− 2.92/− 1.39)	− 0.97 (− 1.48/− 0.58)	− 0.72 (− 1.36/− 0.26)	− 0.12 (− 0.65/0.38)

All records were centered to their mean. 95% confidence levels were calculated using bootstrap methods.

Figure S4 compares coral oxygen isotope records^19,29^ from the IODE region (see Fig. S3 for their locations) with SSTs inferred from Enggano Sr/Ca. While the oxygen isotope records inflate local SST variability, including pIOD induced cooling^29^, they provide valuable records of pIOD occurrence and strength in units of standard deviations^21^. The oxygen isotope record from South Pagai is closest to Enggano island, and derives from an optimal site for IOD reconstruction^29^. Like Enggano, South Pagai suggests that 1972 was not an extreme pIOD event. All coral records thus indicate that extreme pIOD events occurred in 1961, 1963, 1967, 1994, 1997 and 2006 (and 2019, which is not covered by the corals).

### Assessing the significance of discrepancies during extreme pIOD events

**Figure 6:**
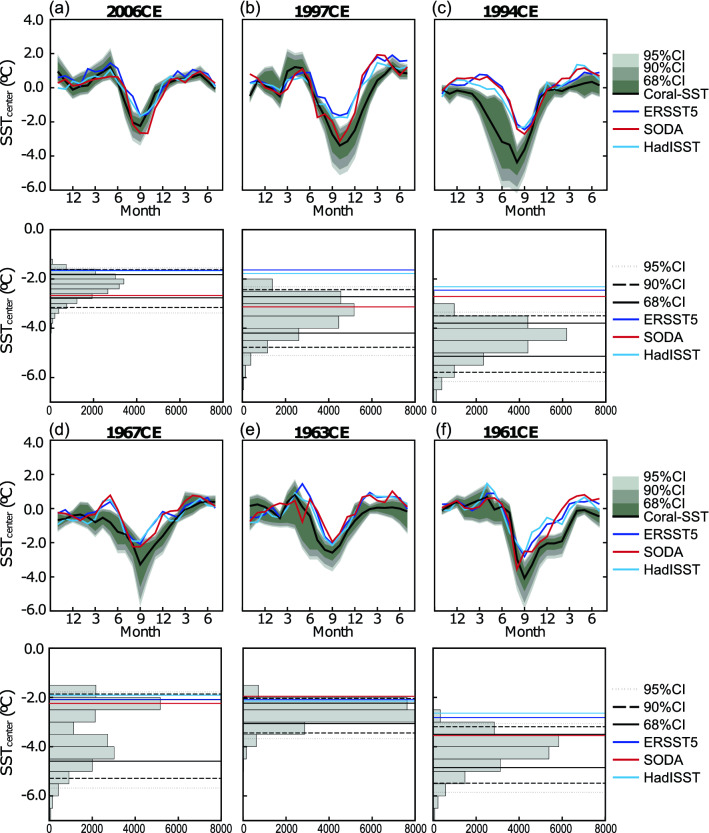
Time series of monthly Enggano SSTs and its uncertainty (black lines and gray shading, upper panel) and histogram of Enggano SSTs estimated from a Monte Carlo simulation that accounts for proxy error for the peak of the pIOD events of 2006, 1997, 1994, 1967, 1963 and 1961. Enggano SST is compared with ERSST5 (dark blue), SODA2 (dark red), and HadISST1 (light blue). Coloured lines on the histograms of Enggano SST are the SST minima in each pIOD year (ERSST5: dark blue; SODA2: dark red; HadISST1: light blue). Thick, dashed, and dotted lines are 68, 90, and 95 percentiles of the Enggano SST minima during pIOD years. See text for discussion.

As shown in Fig. 2, the uncertainties of Enggano SSTs are largest during extreme pIOD events and exceed 2 °C. This is due to the slope uncertainty of the Sr/Ca-SST relationship, which is a factor in the error equation so that the uncertainty of SST anomalies inferred from coral Sr/Ca ratios increases with the magnitude of the Sr/Ca anomaly. We therefore estimate the statistical significance of the observed differences in cooling inferred from the Enggano Sr/Ca record and historical SST products (SODA 2.2.4, ERSST5 and HadISST1) during the pIOD events of 1961, 1963, 1967, 1994, 1997 and 2006 using a Monte Carlo approach^23,30^ (Fig. 6). We find that the differences between the coral SSTs, ERSST5 and HadISST1 are statistically significant during the strongest extreme pIOD events (1961, 1994 and 1997), despite the fact that the uncertainties of the coral SSTs are largest in these extreme years. During the slightly weaker extreme pIOD events of 1963 and 2006, the differences are not significant at the 90% level, although ERSST5 and HadISST1 SSTs are above the upper 68% percentiles of the simulated Enggano SSTs. 1967 is a special case: during this event, the two single coral Sr/Ca records that are combined in the Enggano SST record show large discrepancies (Fig. 6), with KN2 indicating strong, and PB indicating moderate cooling (Fig. 2). This is reflected in the bimodality of the SST distribution in the histogram. Hence, the Enggano coral SSTs are not significantly different from ERSST5 and HadISST1. SODA SSTs generally agree better with the coral SSTs and are only significantly different (too warm) during the extreme pIOD of 1994.

In summary, the strong cooling indicated by the Enggano SST record during the most extreme pIOD events is significantly different from historical SST products, despite the large uncertainties of the coral-based SSTs of more than 2 °C in these years. We therefore conclude that the magnitude of cooling in the IODE region during extreme pIOD events was larger than indicated in historical SST products based on spatial interpolation from sparse observations. The cooling seen during the extreme pIOD of 1961 was comparable in magnitude to the cooling seen in 1994 and 1997, while the 1963 pIOD is more comparable to the 2006 event. A better evaluation of the 1967 pIOD would require additional replication cores due to the deviations between KN2 and PB during this particular event. The oxygen isotope record from South Pagai^29^ suggests that the 1961 event was stronger than 1963, while 1963 was stronger than 1967 (Fig. S4). Hence, Sr/Ca records from South Pagai could help to solve this uncertainty.

## Implications and conclusions

Extreme pIOD events have devastating climatic impacts in the countries surrounding the Indian Ocean, which are home to more than 50% of the world’s population. Understanding the frequency of these extreme events is therefore of primary importance, yet the historical record of SSTs is compromised by sparse observations prior to 1982, and does not adequately capture the non-linear dynamics in the IODE region^11^ so that the magnitude of extreme pIOD events is underestimated. Corals simply mirror ambient water temperature, and are therefore capable of capturing the magnitude of extreme pIOD events more realistically in time periods lacking satellite data. Our results show that coral reconstructions of IODE SSTs can provide additional constraints on the magnitude of extreme pIOD events, and in combination with reanalysis products of historical SSTs that include ocean dynamics, improve our understanding of their variability. Further efforts to expand SST reanalysis products to historical time periods would be important. In addition, the uncertainties of SST reconstructions based on coral Sr/Ca ratios should be reduced, for example by developing further replication cores from the IODE region and/or by reducing the uncertainty of the Sr/Ca-SST relationship by improving proxy calibration. Coral proxy data likely provide the best estimate of IOD variability on historical and millennial time scales^19,21^.

## Methods

### Analytical procedures

In September 2008, two modern *Porites* corals (KN2 and PB) were collected from the fringing reefs of Enggano Island (Fig. S3), in approximately 5 m water depth using a pneumatic drill powered by scuba tanks. After drilling, the cores were cut into 5 mm-thick slabs, x-rayed and prepared for subsampling following standard procedures^31^ (Figs. S5, S6). The cores were subsampled for Sr/Ca analysis at 1 mm intervals, i.e. at approximately monthly resolution. Core KN2 extends from 1930 to 2008 AD, and core PB extents from 1928 to 2008 AD. We used 0.1–0.2 mg of coral powder for Sr/Ca analysis. Sr/Ca ratios were measured at Kiel University using a Spectro Ciros CCD SOP inductively coupled plasma optical emission spectrometer (ICP-OES). Elemental emission signals were simultaneously collected and subsequently processed following a combination of techniques described by^32,33^. Average analytical precision of Sr/Ca measurements as estimated from sample replicates was ~ 0.08% RSD or approximately 0.1 °C. All coral Sr/Ca ratios were normalized to an in-house standard calibrated against JCp-1 (8.838 mmol/mol^34^). Measurements of JCp-1 had a median of 8.832, and a standard deviation of 0.009 (1 sigma) or 0.10% RSD.

The chronology of the coral Sr/Ca records is developed using anchor points following^22^: we assigned September to the Sr/Ca maxima (on average the coldest month) and May to the Sr/Ca minima (the warmest month) in any given year. The data is then linearly interpolated to 12 monthly values per year.

The monthly coral Sr/Ca records were centred by removing their mean and converted to SST units using a coral Sr/Ca-temperature relationship of − 0.06 ± 0.01 mmol/mol per 1 °C^17,23^. The two coral records were then averaged by calculating their arithmetic mean to produce a composite coral SST record^18,35^. The uncertainties of this coral temperature record were calculated using a Monte Carlo approach based on an R script developed by^23^ and expanded in^24^, and include the analytical uncertainties of the Sr/Ca measurements (0.08%RSD for monthly values), the calibration uncertainty of the Sr/Ca-SST slope (± 0.01 mmol/mol per 1ºC^23^), and the difference between the monthly Sr/Ca ratios of the two coral cores (see^24^ for methodology).

### Instrumental and reanalysis data of SST

We use five different ocean temperature products with monthly resolution to compare SST variability and asymmetry in the IODE region:

Optimal interpolation sea surface temperature version 2. (OISST) is constructed using an Optimum Interpolation technique^36^, has a spatial resolution of 1° × 1° grids and extends back until November 1982. OISST is based on satellite data blended with in situ observations from ships and buoys. This product is regarded as the most accurate of observations available. The presence of high variability of IODE SST and strong non-linearity of pIOD events confirm that these properties exist in the real world^11^.

Furthermore, we use two reanalysis products that involve an ocean model in their construction: Simple Ocean Data Assimilation version 3.3.1 (SODA3^37^) is a reanalysis based on the Modular Ocean Model version 5, with a horizontal resolution of 0.5° × 0.5° and a vertical resolution of 50‐levels. SODA 3.3.1 is forced by Modern‐Era Retrospective analysis for Research and Applications Version 2, covering the period of 1980–2015. The observations used in SODA3 include the World Ocean Database of historical hydrographic profiles, in‐situ SST from the International Comprehensive Ocean‐Atmosphere Data Set (ICOADS), and satellite data. We use the potential temperature at 5 m to represent SST in the IODE region (following^11^).

SODA version 2.2.4^25^ is an older version of SODA, but extends back until 1871. The ocean model is based on POP version 2.0.1 numerics with an average horizontal resolution of 0.4° × 0.25° and with 40 levels in the vertical. The atmospheric reanalysis includes only surface observations of synoptic pressure and monthly SST and sea ice distribution from the HadISST1 data set^38^ and uses a data assimilation methodology called the ensemble filter, which relies on a model similar to that used in the original National Center for Environmental Prediction/National Center for Atmospheric Research reanalysis with a 192 × 94 horizontal Gaussian grid. SODA 2.2.4 is mapped onto a uniform global 0.5° × 0.5° horizontal grid. We use the potential temperature at 5 m to represent SST in the IODE region (following^11^).

The National Centers for Environmental Prediction Global Ocean Data Assimilation System (GODAS)^39^ is a reanalysis based on a quasi‐global configuration of the Modular Ocean Model version 3 with a horizontal resolution of 1° × 1°, enhanced to 1/3° in latitude within 10° of the equator, and 40 vertical levels, beginning from 1980. GODAS has assimilated observations from the Tropical Atmosphere Ocean TRITON and PIRATA mooring and Argo profiling floats etc. In this study, the potential temperature at 5 m is used to represent the SST (following^11^).

The two products that do not involve an ocean model in their construction are:

The Extended Reconstructed Sea Surface Temperature Version 5 (ERSST5^40^), which is based on ICOADS SSTs and has a horizontal resolution of 2° × 2° starting from 1854. ERSST5 is constructed as the sum of low‐frequency (LF) and high‐frequency (HF) components. The LF was nonparametrically analyzed using averaging and filtering data over a spatial‐temporal region, while the HF was analyzed by fitting the observed HF anomalies (residual anomalies after subtracting the LF components) to a set of large‐scale spatial‐covariance modes. Satellite data are not included in ERSST5.

The Hadley Center Global Sea Ice and Sea Surface Temperature Version 1.1 (HadISST^38^) is a SST analysis built on an EOF‐based Reduced Space Optimal Interpolation technique, using SST observations from ICOADS, the Met Office Marine Data Bank, and satellite products from 1982 onward. Noninterpolated observed SST anomalies were then superimposed onto the reconstructed SST to improve the localized variability. HadISST has a horizontal resolution of 1° × 1° and starts from 1870.

### Extreme pIOD events

In the satellite era, 1994, 1997 and 2006 are taken as extreme pIOD events following^11^.

On historical time scales, the detection of pIOD events depends on the instrumental SST product used, and there are notable differences between various studies (see^28^ for a discussion). In this study, we investigate the ‘true’ pIOD events identified in^28^, i.e. 1946, 1961, 1963, 1967, 1972. We estimate the magnitude of cooling in the IODE region from the Enggano Sr/Ca record and classify the event as ‘extreme pIOD’ when it is ≥ 2006.

## Supplementary Information

Supplementary Information.

## Data Availability

The data used in this study can be downloaded from the following websites: OISST (https://psl.noaa.gov/data/gridded/data.noaa.oisst.v2.html); SODA (https://www2.atmos.umd.edu/~ocean/index_files); GODAS (https://psl.noaa.gov/data/gridded/data.godas.html); ERSST5 (https://psl.noaa.gov/data/gridded/data.noaa.ersst.v5.html); HadISST1 (https://www.metoffice.gov.uk/hadobs/hadisst/). The coral datasets generated during the current study are available in the National Centers for Environmental Information Paleoclimatology repository [https://www.ncei.noaa.gov/products/paleoclimatology].
