# Supplementary material for: Coral Sr/Ca records provide realistic representation of eastern Indian Ocean cooling during extreme positive Indian Ocean Dipole events
Source: Sci Rep. 2022 Jun 23;12:10642. doi: 10.1038/s41598-022-14617-9 (PMC9226043; doi:10.1038/s41598-022-14617-9)
Supplement: Supplementary file 1 — Supplementary Information. [file 41598_2022_14617_MOESM1_ESM.docx]

**Supplementary Tables**

Table S1: Correlation matrix of annual mean coral SST and various SST products centered at Enggano Island (5°S, 102°E) for the time period 1982-2008 (n=26). All correlations are statistically significant (p<0.01, *p<0.05).

|  | **PB** | **KN2** | **Enggano** | **OISST** | **SODA3** | **GODAS** | **ERSST5** | **HadISST1** |
| --- | --- | --- | --- | --- | --- | --- | --- | --- |
| **PB** | x | 0.81 | 0.95 | 0.76 | 0.78 | 0.45* | 0.68 | 0.66 |
| **KN2** |  | x | 0.94 | 0.76 | 0.8 | 0.61 | 0.59 | 0.75 |
| **Enggano** |  |  | x | 0.81 | 0.81 | 0.61 | 0.65 | 0.72 |
| **OISST** |  |  |  | x | 0.95 | 0.77 | 0.86 | 0.9 |
| **SODA3** |  |  |  |  | x | 0.83 | 0.87 | 0.86 |
| **GODAS** |  |  |  |  |  | x | 0.83 | 0.68 |
| **ERSST5** |  |  |  |  |  |  | x | 0.83 |
| **HadISST1** |  |  |  |  |  |  |  | x |

Table S2: Correlation matrix of annual mean coral SST and various historical SST products centered at Enggano Island (5°S, 102°E) for the time period 1930-2008 (regular, n=77) and 1958-2008 (bold, n=50). All correlations are statistically significant (p<0.01).

|  | **PB** | **KN2** | **Enggano** | **SODA2.2.4** | **ERSST5** | **HadISST1** |
| --- | --- | --- | --- | --- | --- | --- |
| **PB** | x | 0.58 | 0.87 | 0.53 | 0.53 | 0.54 |
| **KN2** | **0.74** | x | 0.77 | 0.61 | 0.58 | 0.64 |
| **Enggano** | **0.93** | **0.93** | x | 0.63 | 0.61 | 0.54 |
| **SODA2.2.4** | **0.67** | **0.7** | **0.72** | x | 0.88 | 0.88 |
| **ERSST5** | **0.69** | **0.66** | **0.69** | **0.87** | x | 0.87 |
| **HadISST1** | **0.69** | **0.75** | **0.78** | **0.87** | **0.87** | x |

**Supplementary Figures**

*Fig. S1:* Skewness of September-November mean SSTs in the Indian Ocean as portrayed in various SST products for the time period 1982-2008. The location of Enggano Island is marked by a red x. Charts computed at the knmi climate explorer (<https://climexp.knmi.nl>; date accessed 14/03/2022). **
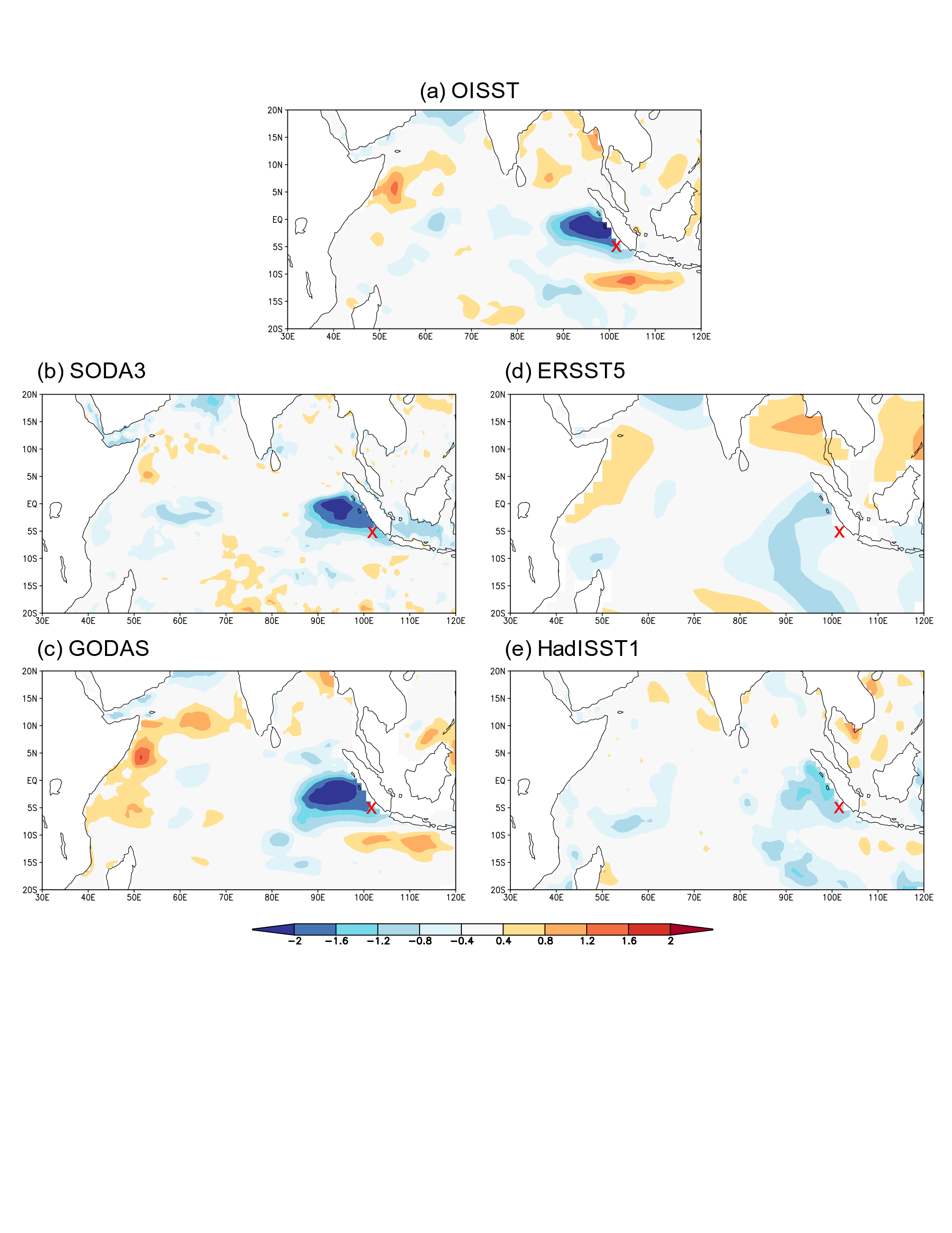
**

*Fig. S2:* Standard deviation of September-November mean SSTs in the Indian Ocean as portrayed in various SST products for the time period 1982-2008. The location of Enggano Island is marked by a green x. Charts computed at the knmi climate explorer (<https://climexp.knmi.nl>; date accessed 14/03/2022).

**
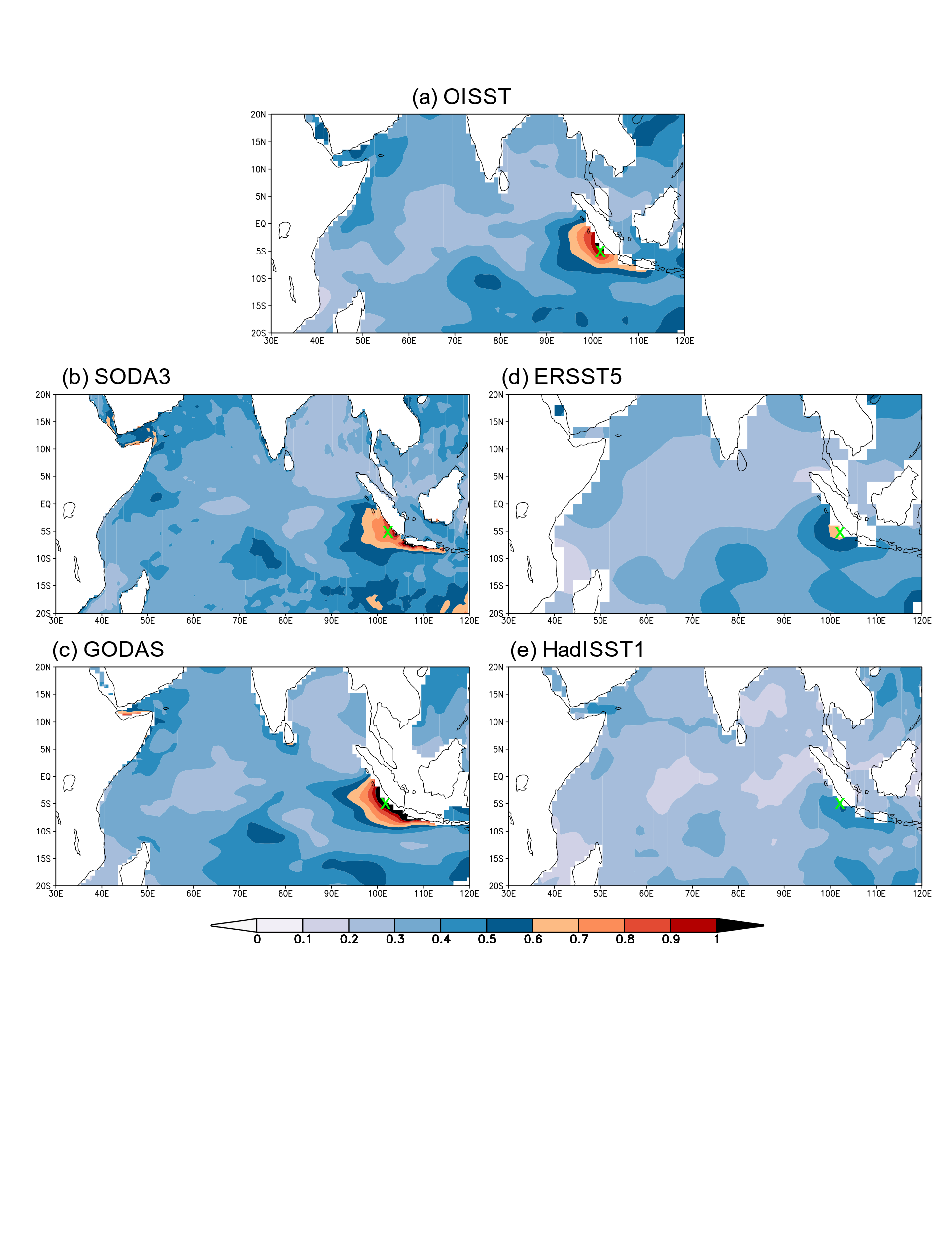
**

*Fig. S3:* Left: Location of Enggano Island off the coast of Sumatra (Indonesia) (black arrow and rectangle). Arrows indicate location of coral oxygen isotope records from South Pagai and Mentawai published in [1,2]. Right: Enggano Island and location of coral cores. Charts computed at the knmi climate explorer and Google Earth, respectively. Date accessed: 9/3/2022.

*Fig. S4:* Monthly time series of coral oxygen isotope records from Mentawai (a) and South Pagai (b) compared with the Enggano coral Sr/Ca record. All proxies records are converted to SST anomalies according to their temperature dependence (-0.2 permil per 1°C for oxygen isotopes and -0.06 mmol/mol per 1°C for Sr/Ca ratios). White arrows mark positive IOD events identified in [3,4] (see Methods for discussion). Grey arrow marks the 1982/83 El Nino. Note that the South Pagai and Enggano records show similar variability, although South Pagai overestimates the cooling during extreme pIOD events, indicating SST-covariant changes in δ^18^O seawater.

**

*Fig. S5:* X ray images of core PB showing sampling transects (red lines) and annual density bands. Note the goodness of fit between slabs.


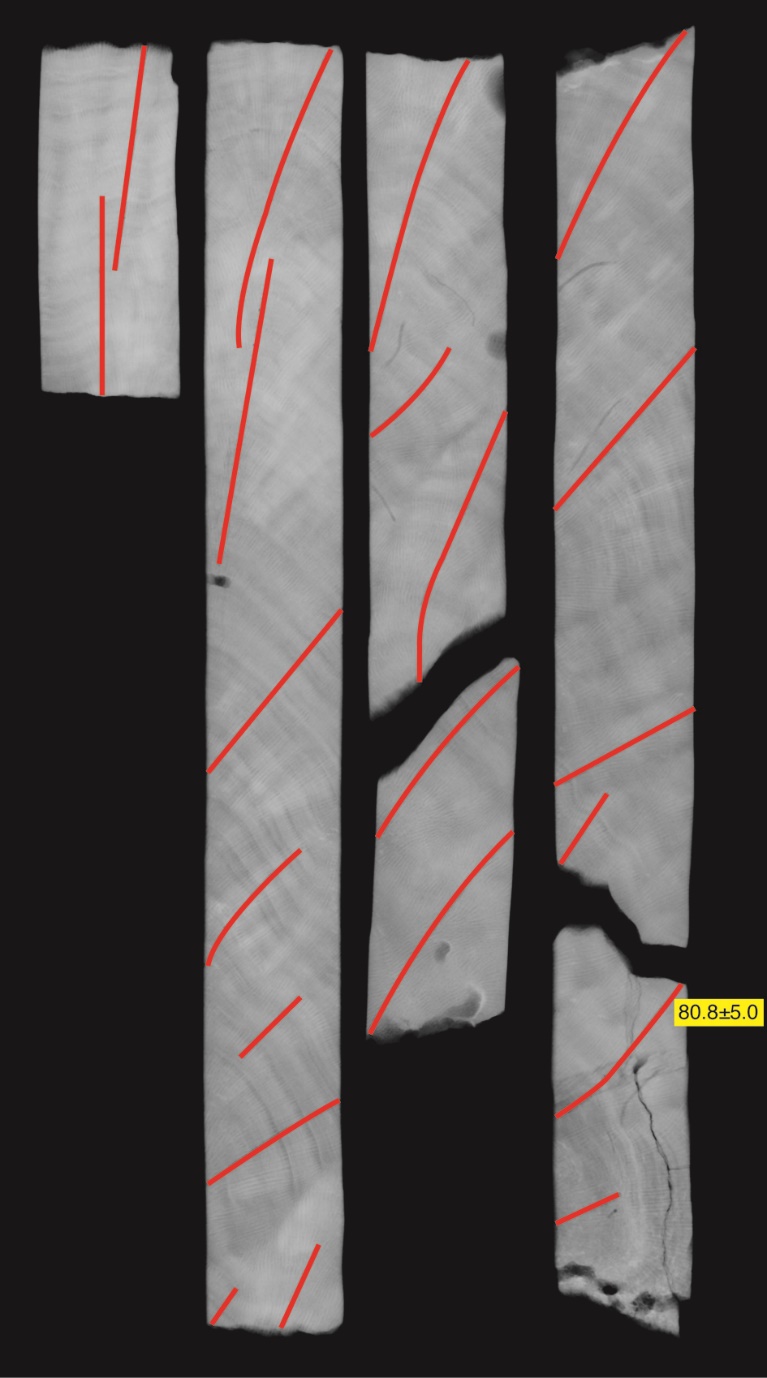


*Fig. S6:* X ray images of core KN2 showing sampling transects (red lines). Annual density bands are not very clear so that the age model relies on the seasonality of the trace element data. Note the goodness of fit between slabs.


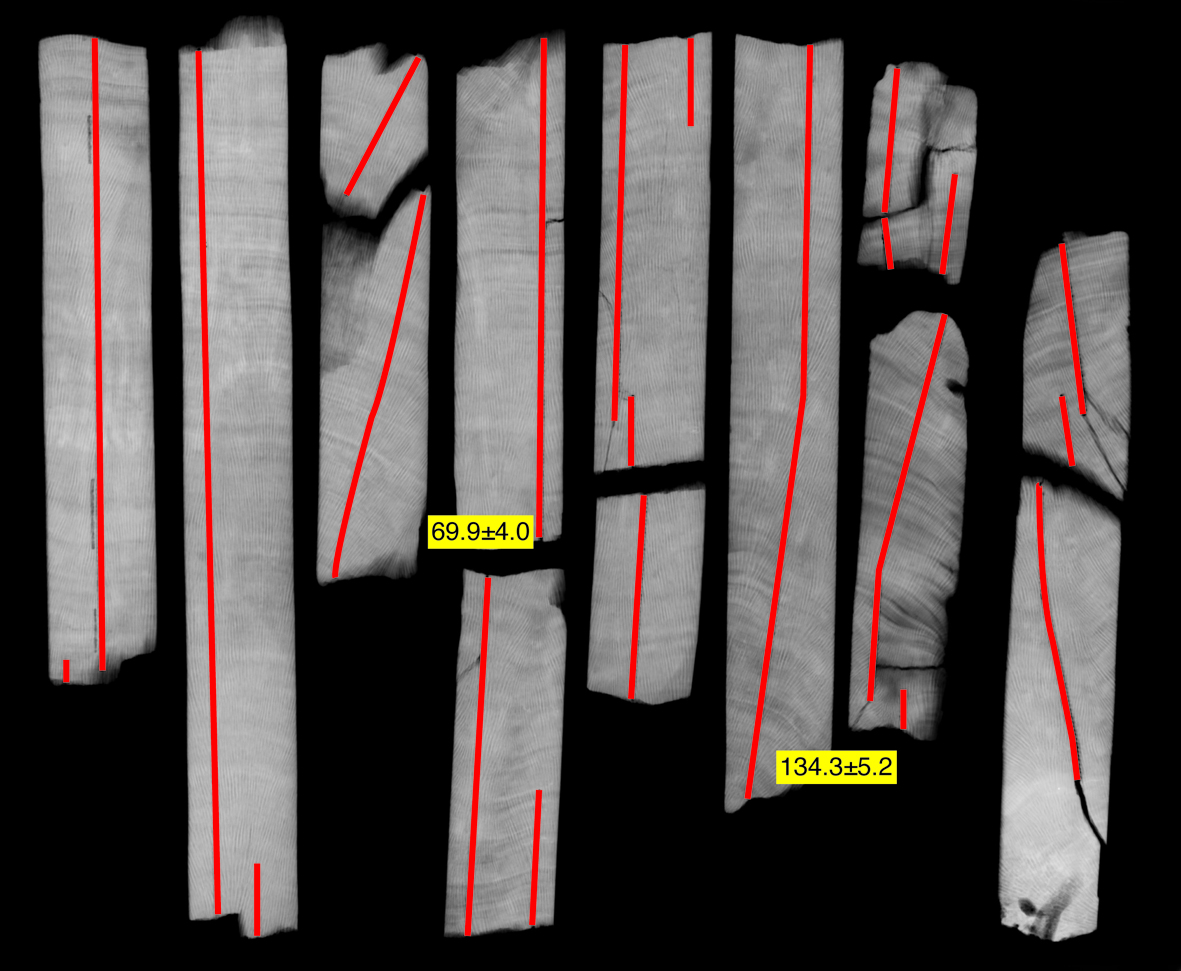


References

1. Abram, N. J. *et al.* Optimized coral reconstructions of the Indian Ocean Dipole: An assessment of location and length considerations. *Paleoceanography* **30,** 1391–1405 (2015).

2. Abram, N. J., Gagan, M. K., Cole, J. E., Hantoro, W. S. & Mudelsee, M. Recent intensification of tropical climate variability in the Indian Ocean. *Nat. Geosci.* **1,** 849–853 (2008).

3. Yang, K. *et al.* Oceanic Processes in Ocean Temperature Products Key to a Realistic Presentation of Positive Indian Ocean Dipole Nonlinearity. *Geophys. Res. Lett.* **47** (2020).

4. Verdon-Kidd, D. C. On the classification of different flavours of Indian Ocean Dipole events. *Int J Climatol* **38,** 4924–4937 (2018).
